# The Prevalence and Consequence of Depression After Bariatric Surgery Among Adults in Saudi Arabia

**DOI:** 10.7759/cureus.59945

**Published:** 2024-05-09

**Authors:** Osman Suliman, Muhanad M Esailan, Abdulaziz M Alraddadi, Aos S Shaker, Linah A Alsharif, Meshal S Aljohani, Rema F Alharbi, Fadi A Almohammadi, Ghadeer A Alahmadi, Samah Osailan

**Affiliations:** 1 Clinical Sciences, College of Medicine, Al-Rayan Colleges, Al Madinah, SAU; 2 Medicine and Surgery, Al-Rayan Colleges, Al Madinah, SAU; 3 General Surgery, Faculty of Medicine, King Abdulaziz University, Jeddah, SAU

**Keywords:** psychiatry, saudi arabia, obesity, bariatric surgery, depression

## Abstract

Background: Bariatric surgery is a commonly performed group of procedures for individuals with severe obesity, and its impact on mental health, particularly depression, has gained significant attention. In Saudi Arabia, the prevalence and consequences of depression following bariatric surgery among adults is an area of growing concern. The study aimed to assess the prevalence of depression after bariatric surgery and estimate the consequences of depression after bariatric surgery.

Methods: A cross-sectional study was conducted among adults in Saudi Arabia who had undergone bariatric surgery. The participants were recruited from different bariatric surgery centers across the country. The data was collected using a self-administered questionnaire that had three main sections, including demographic information, clinical characteristics, and the Patient Health Questionnaire-9 (PHQ-9) to assess depression.

Results: The results of the study showed that among adults in Saudi Arabia who had undergone bariatric surgery, the prevalence of depression was high. Of the participants, 23.5% were found to have depression after bariatric surgery. This high prevalence suggests that depression is a significant consequence of bariatric surgery.

Conclusion: A high number of adults in Saudi Arabia experience depression after bariatric surgery. This shows a need for mental health checks and support before and after the surgery. With mental healthcare as a regular part of bariatric programs, patients would have a better chance of success and overall well-being. More research is needed to understand why depression happens after surgery and how to prevent it.

## Introduction

Depression is a common concern among individuals undergoing bariatric surgery. Studies have shown varying prevalence rates of depression post-bariatric surgery, ranging from 15% to 31.3% [1-3]. Alyahya and Alnujaidi [1] conducted a systematic review and meta-analysis, revealing a prevalence of post-bariatric surgery depression at 15%. Another study reported a depression prevalence of 31.3% post-bariatric surgery [3]. A study by Alshammari et al. [4] at King Khalid University Hospital in Riyadh reported varying levels of depression post-bariatric surgery, with 8.2% of patients showing moderately high depression levels and 4.4% experiencing severe depression. While depression has been reported to improve after bariatric surgery in many cases [5,6], according to Ivezaj and Grilo (2014), there are instances where some patients may experience worsening depressive symptoms following the procedure. Factors such as adherence to lifestyle recommendations, binge eating, and social support have been identified as predictors of weight loss post-surgery and may also influence depression outcomes [7].

Preoperative depression has been associated with increased severity of medical comorbidities in bariatric surgery candidates and may impact postoperative weight loss [8]. Psychological evaluations conducted before surgery often include screening for depression, as it is considered an integral part of the assessment process [9,10]. Additionally, studies have highlighted the importance of providing psychological support both before and after bariatric surgery, especially considering the complex psychological histories of many patients, which may include depression, anxiety, poor self-esteem, and eating disorder symptoms [10]. Another study by Mitchell et al. [11] highlighted the frequent occurrence of depressive symptoms among severely obese adults, emphasizing the importance of addressing psychological well-being in this population. While some research suggests that bariatric surgery can lead to beneficial effects on depressive symptoms, with improvements peaking around 6-12 months post-operation [12,13], there are also cases where the benefits may be transient, and depressive symptoms could regress over time [12]. It is crucial for healthcare providers to monitor patients for changes in depressive symptoms post-bariatric surgery and provide appropriate support and interventions as needed to ensure optimal mental health outcomes alongside the physical benefits of the procedure.

Thus, the prevalence and consequences of depression after bariatric surgery among adults are multifaceted, necessitating a comprehensive approach to patient care that addresses mental health alongside surgical outcomes. Understanding the complexities of postoperative mental health challenges is crucial for optimizing patient well-being and long-term success following bariatric surgery.

## Materials and methods

Study design and population

A cross-sectional study was conducted using data from adult patients who had undergone bariatric surgery in Saudi Arabia. The study aimed to assess the prevalence and consequences of depression in this population. Participants were selected from multiple bariatric surgery centers across the country using a systematic random sampling method. Various validated mental health assessment tools were utilized to screen for depression, and demographic and clinical data were collected to analyze the potential risk factors associated with depression post-surgery. The inclusion criteria included adult residents of Saudi Arabia who had undergone bariatric surgery, regardless of gender, and were 18 years old or older. Conversely, the exclusion criteria encompassed non-Saudi Arabian residents with no history of bariatric surgery, as well as Saudi Arabian residents who refused to participate in the study and were under the age of 18.

Sample size

In Saudi Arabia, there are more than 20,000 bariatric surgeries that take place every year [14]. Considering this rate of bariatric surgeries and for a 95% confidence interval (CI), the sample size was determined to be 377 using Raosoft software for sample size calculation (http://www.raosoft.com/samplesize.html).

Data collection tool and procedure

The researchers used a convenience sampling technique to select participants for the study. The sample size for the study was determined to be 377 using Raosoft software for sample size calculation, considering a confidence level of 95% and an estimated rate of bariatric surgeries in Saudi Arabia. The data was collected through an online questionnaire divided into three sections. The first section gathered demographic information, including sex, age, marital status, educational level, and income. The second section focused on pre-surgery information such as smoking habits, comorbidities, obesity-related health problems, body mass index (BMI) before and after surgery, and time of surgery. The first and second portions of the questionnaire were obtained from the study by Alshammari et al. [4]. The third section utilized the Patient Health Questionnaire-9 (PHQ-9) criteria to assess depression. The PHQ-9 assessment tool consists of a total of nine items, each scored from "0" (absent) to "3" (present nearly every day), resulting in a total possible score ranging from 0 to 27 [15]. The interpretation of PHQ-9 scores was based on the following classification. A PHQ-9 score ranging from 0 to 4 indicates minimal depression, while scores between 5 and 9 signify mild depression. Moderate depression falls within the range of 10-14, whereas moderately severe depression corresponds to scores between 15 and 19. Finally, a PHQ-9 score of 20-27 is indicative of severe depression [15]. PHQ-9 scores were additionally interpreted for diagnosing depression, employing a cutoff value of 10. For a cutoff score of 10, the pooled sensitivity was 0.85 (95% CI: 0.75-0.91), while the pooled specificity stood at 0.89 (95% CI: 0.83-0.92) [16].

Data analysis

Data obtained from this study were analyzed using Statistical Package for the Social Sciences (SPSS) version 26.0 (IBM SPSS Statistics, Armonk, NY) for Windows. Descriptive statistics were computed for all variables included in the study. Categorical variables are presented as frequencies and percentages, while continuous variables are summarized as means ± standard deviations (SD). Chi-square tests were used to examine the distribution of perceived depression categories (minimal, mild, moderate, moderately severe, and severe) across different levels of each categorical predictor variable, including sex, age group, marital status, educational level, occupation/employment status, household's monthly income in Saudi Riyals (SAR), smoking habit, comorbidity, history of obesity-related health problems, esophageal regurgitation, body mass index level prior to bariatric surgery, BMI level post-bariatric surgery, BMI improvement post-bariatric surgery, type of bariatric surgery received, time of bariatric surgery, and whether the patients experienced any bariatric surgical-related problems/complications. Statistical significance was set at p < 0.05. The chi-square test was used to find the association between bariatric surgery and the presence or absence of depression among adults.

Ethical considerations

Ethical approval for the study was obtained from the Al-Rayan Research Ethics Committee, registered with the National Bioethics Committee in King Abdulaziz City for Science and Technology (KACST), Saudi Arabia (reference number: HA-03-M-122-076). Online consent was taken before participation in the study before completing the online questionnaire. All personal data was handled with strict confidentiality.

## Results

The study sample comprised 366 participants, with a higher representation of females (63.93%) compared to males (36.07%). Regarding age distribution, participants were varied, with the largest proportion falling within the age group of less than 30 years (39.07%), followed by those aged 30-39 years (27.60%), 40-49 years (23.77%), and finally, participants aged 50 years or older (9.56%). In terms of marital status, the majority of participants were married (64.21%), while the remaining proportion was unmarried (35.79%). Educational backgrounds varied among participants, with the majority (68.03%) holding a university degree, followed by those with high school or less education (14.75%), diploma degree (11.48%), and master's and PhD degrees (5.74%). Participants' employment status showed diversity, with a notable proportion being employed in the governmental sector (41.80%), followed by those who were unemployed or housewives (33.06%), and those employed in the private sector (25.14%). Household monthly income in Saudi Riyals was spread across different brackets, with a significant portion falling within the range of 5,000-10,000 SAR (28.42%), followed by 10,000-20,000 SAR (27.60%), less than 5,000 SAR (27.87%), and greater than 20,000 SAR (16.12%). In terms of smoking habits, the majority of participants were nonsmokers (64.48%), followed by smokers (24.59%), and ex-smokers (10.93%). This is shown in Table 1.

**Table 1:** Sociodemographic characteristics of the patient cohort The values are presented in numbers (%). SAR: Saudi Riyal

Sociodemographic characteristics		Count (number (%))
Sex	Male	132 (36.07%)
	Female	234 (63.93%)
Age group	<30 years	143 (39.07%)
	30-39 years	101 (27.60%)
	40-49 years	87 (23.77%)
	≥50 years	35 (9.56%)
Marital status	Unmarried	131 (35.79%)
	Married	235 (64.21%)
Level of education	High school or less education	54 (14.75%)
	Diploma degree	42 (11.48%)
	University degree	249 (68.03%)
	Master's and PhD degrees	21 (5.74%)
Occupation/employment	Unemployed/housewife	121 (33.06%)
	Employed (private sector)	92 (25.14%)
	Employed (governmental sector)	153 (41.80%)
Household income	<5,000 SAR	102 (27.87%)
	5,000-10,000 SAR	104 (28.42%)
	10,000-20,000 SAR	101 (27.60%)
	>20,000 SAR	59 (16.12%)
Smoking habit	Nonsmoker	236 (64.48%)
	Ex-smoker	40 (10.93%)
	Smoker	90 (24.59%)

A comprehensive examination of obesity-related health problems and bariatric surgery details among participants yielded insightful findings. Regarding obesity-related health problems, a significant proportion of participants (51.37%) reported experiencing sleep breathing disorders, with the majority (51.37%) noting improvement post-surgery. In terms of esophageal reflux, 39.07% of participants reported its presence, with notable improvements post-surgery observed in 39.07% of cases. When considering the distribution of participants across body mass levels before obesity surgery, the majority fell within the 30%-35% range (47.81%), followed by 35%-40% (31.42%), and >40% (20.77%). Postoperatively, most participants achieved body mass levels within the 20%-25% range (33.06%). Moreover, the vast majority (93.72%) reported improvement in body mass index following surgery. Sleeve gastrectomy surgery emerged as the predominant surgical intervention (85.52%), followed by gastric bypass surgery (14.48%). Timing-wise, a substantial portion of participants underwent surgery 1-2 years prior (25.41%). Despite the effectiveness of bariatric surgery, a notable proportion (21.04%) reported experiencing problems or complications post-surgery. These insights shed light on the complexities of bariatric surgery and underscore the importance of considering individual variations in surgical outcomes when assessing their potential impact on perceived depression scores. This information is shown in Table 2.

**Table 2:** Descriptive analysis of obesity-related questions The values are presented in numbers (%).

Obesity-related		Count (number (%))
History of obesity-related health problems (sleep breathing disorder)	Yes, but had improved and disappeared post-surgically	188 (51.37%)
	Yes, but unchanged compared to the preoperative time	24 (6.56%)
	No, I did not complain of disturbance during sleep	154 (42.08%)
Esophageal reflux	Yes, but had improved and disappeared post-surgically	143 (39.07%)
	Yes, but unchanged compared to the preoperative time	60 (16.39%)
	Yes, worsened	28 (7.65%)
	No, I did not complain of esophageal reflux	135 (36.89%)
Body mass level before obesity surgery	30%-35%	175 (47.81%)
	35%-40%	115 (31.42%)
	>40%	76 (20.77%)
Postoperative body mass level for obesity	<20%	105 (28.69%)
	20%-25%	121 (33.06%)
	25%-30%	71 (19.40%)
	30%-35%	53 (14.48%)
	35%-40%	16 (4.37%)
Improving body mass index after surgery for obesity	Not improved	23 (6.28%)
	Improved	343 (93.72%)
Type of bariatric surgery performed	Sleeve gastrectomy surgery	313 (85.52%)
	Gastric bypass surgery	53 (14.48%)
When was bariatric surgery performed	<6 months	77 (21.04%)
	6 months-1 year	73 (19.95%)
	1-2 years	93 (25.41%)
	2-3 years	46 (12.57%)
	3-4 years	21 (5.74%)
	>4 years	56 (15.30%)
Did you experience any problems/complications related to bariatric surgery? (n = 360)	No	283 (77.32%)
	Yes	77 (21.04%)

An analysis of anxiety-related variables revealed several key findings among bariatric surgery patients. The majority of participants (78.42%) did not visit a psychiatrist's office before surgery, while 21.58% reported having visited one. Among those who did visit a psychiatrist, the timing of visits varied, with 9.02% unable to recall the timing, 6.56% visiting less than a year before surgery, 3.28% visiting 1-3 years before surgery, and 2.73% visiting four years before surgery. A minority of participants (12.84%) reported being diagnosed with a mental illness before surgery, with panic attacks and social phobia (2.73%), personality disorders (3.01%), and other psychiatric illnesses (4.10%) being the most commonly reported diagnoses. Additionally, 19.40% of participants reported having close family members diagnosed with a psychological/mental illness, with schizophrenia (5.19%) and panic attacks (4.92%) being the most prevalent diagnoses among family members. Regarding substance use, the majority of participants (93.72%) reported not using medications or illegal drugs before surgery, while 6.28% reported using them frequently. Furthermore, about half of the participants (51.37%) reported encountering recent family, social, economic, or work problems. This is shown in Table 3.

**Table 3:** Descriptive analysis of anxiety-related questions The values are presented in numbers (%).

Anxiety		Count (number (%))
Did you visit a psychiatrist's office before surgery?	No	287 (78.42%)
	Yes	79 (21.58%)
If the answer is yes, when do you visit the psychiatrist’s clinic? (n = 79)	Visited but cannot remember when	33 (41.77%)
	Yes, in less than one year	24 (30.38%)
	Yes, 1-3 years before	12 (15.19%)
	Yes, four years before surgery	10 (12.66%)
Have you been diagnosed with any mental illness?	No	319 (87.16%)
	Yes	47 (12.84%)
If the answer is yes, what psychological diagnoses have you been diagnosed with? (n = 47)	Panic attacks and social phobia	10 (21.28%)
	Personality disorders	11 (23.40%)
	Other psychiatric illness	15 (31.91%)
	Obsessive disorder	7 (14.89%)
	Schizophrenia	4 (8.51%)
Are there any close family members who have been diagnosed with a psychological/mental illness?	No	295 (80.60%)
	Yes	71 (19.40%)
If the answer is yes, what psychiatric diagnosis do they have? (n = 71)	Panic attacks	18 (25.35%)
	Other psychiatric illness	17 (23.94%)
	Personality disorders	6 (8.45%)
	Obsessive-compulsive disorder	11 (15.49%)
	Schizophrenia	19 (26.76%)
Have you used medications or illegal drugs before?	No	343 (93.72%)
	Yes, frequently	23 (6.28%)
Have you recently encountered family, social, economic, or work problems?	No	178 (48.63%)
	Yes	188 (51.37%)

The mean PHQ-9 score among participants was found to be 6.80 ± 4.96. Scores ranged from a minimum of 0 to a maximum of 27. This suggests that, on average, participants reported experiencing mild to moderate levels of depression symptoms, as scores below 5 typically indicate minimal or no depression, scores between 5 and 9 suggest mild depression, scores between 10 and 14 indicate moderate depression, and scores above 15 indicate severe depression. The variability in scores, as indicated by the standard deviation, suggests that there was some diversity in the severity of depression symptoms among participants. This is shown in Table 4. 

**Table 4:** Descriptive analysis of PHQ-9 scores PHQ-9: Patient Health Questionnaire-9, SD: standard deviation

PHQ-9 score	Mean ± SD	6.8 ± 4.96
Severity of depression (number (%))	Minimal	117 (31.97%)
	Mild	163 (44.54%)
	Moderate	61 (16.67%)
	Moderately severe	17 (4.64%)
	Severe	8 (2.19%)
Diagnosis of depression (number (%))	No depression	280 (76.50%)
	Depression	86 (23.50%)

The chi-square tests revealed significant associations between the severity of depression symptoms, as measured by PHQ-9 categories, and various demographic and lifestyle factors among bariatric surgery patients. Females exhibited a higher prevalence of moderate to severe depression compared to males (χ² = 56.389, df = 8, p < 0.001). Younger age groups, particularly those under 30 years old, reported elevated levels of depression severity (χ² = 48.840, df = 12, p < 0.001). Marital status also played a significant role, with unmarried individuals demonstrating higher severity levels compared to married counterparts (χ² = 25.463, df = 4, p < 0.001). While educational attainment did not show a significant association (χ² = 10.097, df = 12, p = 0.607), income level exhibited a significant relationship, indicating that lower household income was linked to increased depression severity (χ² = 29.875, df = 12, p = 0.003). Moreover, smokers displayed higher severity levels compared to non-smokers (χ² = 56.389, df = 8, p < 0.001). These findings underscore the importance of considering demographic and lifestyle factors when assessing and addressing depression among bariatric surgery patients, highlighting the need for tailored interventions that account for these variables. Furthermore, while there was no significant association between the severity of depression and the presence of chronic diseases overall (χ² = 7.799, df = 4, p = 0.099), there was evidence of an association between the severity of depression and specific chronic diseases (χ² = 47.473, df = 24, p = 0.003), indicating potential differences in mental health status based on the type of chronic illness.

Additionally, the analysis revealed that participants who reported a history of obesity-related health problems, such as sleep breathing disorders, displayed varying levels of depression severity (χ² = 17.149, df = 8, p = 0.029). Those who experienced improvements in these health issues post-surgery tended to report lower levels of depression, suggesting a potential link between physical health improvements and mental well-being. Similarly, participants with esophageal reflux showed varying levels of depression severity (χ² = 33.179, df = 12, p < 0.001), with those experiencing worsened reflux post-surgery reporting higher levels of depression. Moreover, the severity of depression symptoms significantly differed across different categories of body mass index (BMI) levels before obesity surgery (χ² = 36.485, df = 8, p < 0.001) and postoperative BMI levels (χ² = 45.096, df = 16, p < 0.001), indicating a potential association between BMI changes and mental health outcomes post-bariatric surgery.

The type and timing of bariatric surgery also exhibited significant associations with depression severity. Participants who underwent sleeve gastrectomy surgery showed varying levels of depression severity compared to those who underwent gastric bypass surgery (χ² = 13.505, df = 4, p = 0.00906), suggesting that different surgical procedures may have differential impacts on mental health outcomes. Similarly, the time passed since surgery showed significant associations with depression severity (χ² = 65.636, df = 20, p < 0.001), with individuals undergoing surgery within the past six months reporting higher levels of depression compared to those with longer postoperative periods. Furthermore, participants who experienced problems or complications related to bariatric surgery tended to report higher levels of depression severity (χ² = 21.504, df = 4, p = 0.000252), highlighting the potential psychological impact of surgical complications.

Additionally, visiting a psychiatrist's office before surgery did not significantly influence depression severity (χ2 = 3.896, df = 4, p = 0.420), suggesting that prior psychiatric consultations may not directly correlate with postoperative depression outcomes. However, the timing of these visits showed a significant association with depression severity (χ2 = 78.082, df = 8, p = 0.000), with participants who visited a psychiatrist within the year preceding surgery reporting higher levels of depression.

Moreover, participants diagnosed with mental illnesses or reporting family members with psychiatric diagnoses exhibited varying levels of depression severity (χ2 = 15.442, df = 4, p = 0.003; χ2 = 15.786, df = 4, p = 0.003, respectively). Those with diagnosed mental illnesses tended to report higher levels of depression, highlighting the importance of addressing comorbid psychiatric conditions in bariatric surgery patients. Similarly, participants with family members diagnosed with psychiatric illnesses showed elevated levels of depression, suggesting potential familial influences on mental health outcomes.

Furthermore, substance use behaviors, including the use of medications or illegal drugs, showed a significant association with depression severity (χ2 = 17.552, df = 4, p = 0.002), with frequent drug users reporting higher levels of depression. Similarly, participants experiencing recent family, social, economic, or work-related problems exhibited elevated levels of depression severity (χ2 = 56.215, df = 4, p < 0.001), indicating the potential impact of psychosocial stressors on mental health outcomes. This is summarized in Table 5.

**Table 5:** Distribution of depression severity by demographic and health factors The values are presented in numbers (%). SAR: Saudi Riyal

Categories		Severity of depression (number (%))					Total	p value (chi-square)
		Minimal	Mild	Moderate	Moderately severe	Severe		
Sex	Male	30 (22.73%)	55 (41.67%)	34 (25.76%)	10 (7.58%)	3 (2.27%)	132 (36.07%)	0
	Female	87 (37.18%)	108 (46.15%)	27 (11.54%)	7 (2.99%)	5 (2.14%)	234 (63.93%)	
Age group	<30 years	20 (13.99%)	75 (52.45%)	33 (23.08%)	11 (7.69%)	4 (2.8%)	143 (39.07%)	0
	30-39 years	46 (45.54%)	33 (32.67%)	18 (17.82%)	3 (2.97%)	1 (0.99%)	101 (27.6%)	
	40-49 years	34 (39.08%)	37 (42.53%)	10 (11.49%)	3 (3.45%)	3 (3.45%)	87 (23.77%)	
	≥50 years	17 (48.57%)	18 (51.43%)	0 (0%)	0 (0%)	0 (0%)	35 (9.56%)	
Marital status	Unmarried	26 (19.85%)	57 (43.51%)	32 (24.43%)	11 (8.4%)	5 (3.82%)	131 (35.79%)	0
	Married	91 (38.72%)	106 (45.11%)	29 (12.34%)	6 (2.55%)	3 (1.28%)	235 (64.21%)	
Level of education	High school or less education	17 (31.48%)	22 (40.74%)	9 (16.67%)	4 (7.41%)	2 (3.7%)	54 (14.75%)	0.607
	Diploma degree	9 (21.43%)	21 (50%)	10 (23.81%)	2 (4.76%)	0 (0%)	42 (11.48%)	
	University degree	83 (33.33%)	111 (44.58%)	41 (16.47%)	9 (3.61%)	5 (2.01%)	249 (68.03%)	
	Master's and PhD degrees	8 (38.1%)	9 (42.86%)	1 (4.76%)	2 (9.52%)	1 (4.76%)	21 (5.74%)	
Occupation/employment	Unemployed/housewife	34 (28.1%)	55 (45.45%)	21 (17.36%)	7 (5.79%)	4 (3.31%)	121 (33.06%)	0.091
	Employed (private sector)	23 (25%)	42 (45.65%)	22 (23.91%)	5 (5.43%)	0 (0%)	92 (25.14%)	
	Employed (governmental sector)	60 (39.22%)	66 (43.14%)	18 (11.76%)	5 (3.27%)	4 (2.61%)	153 (41.8%)	
Income	<5,000 SAR	45 (44.12%)	38 (37.25%)	13 (12.75%)	6 (5.88%)	0 (0%)	102 (27.87%)	0.003
	5,000-10,000 SAR	24 (23.08%)	52 (50%)	18 (17.31%)	5 (4.81%)	5 (4.81%)	104 (28.42%)	
	10,000-20,000 SAR	39 (38.61%)	39 (38.61%)	18 (17.82%)	2 (1.98%)	3 (2.97%)	101 (27.6%)	
	>20,000 SAR	9 (15.25%)	34 (57.63%)	12 (20.34%)	4 (6.78%)	0 (0%)	59 (16.12%)	
Smoking habit	Nonsmoker	102 (43.22%)	95 (40.25%)	28 (11.86%)	6 (2.54%)	5 (2.12%)	236 (64.48%)	0
	Ex-smoker	8 (20%)	22 (55%)	4 (10%)	4 (10%)	2 (5%)	40 (10.93%)	
	Smoker	7 (7.78%)	46 (51.11%)	29 (32.22%)	7 (7.78%)	1 (1.11%)	90 (24.59%)	
Are there any accompanying chronic diseases?	No	83 (32.3%)	107 (41.63%)	44 (17.12%)	15 (5.84%)	8 (3.11%)	257 (70.22%)	0.099
	Yes	34 (31.19%)	56 (51.38%)	17 (15.6%)	2 (1.83%)	0 (0%)	109 (29.78%)	
If the answer is yes, please answer the question (n = 109)	High blood fat/cholesterol levels	0 (0%)	0 (0%)	2 (100%)	0 (0%)	0 (0%)	2 (0.55%)	0.003
	Hypertension	6 (19.35%)	17 (54.84%)	8 (25.81%)	0 (0%)	0 (0%)	31 (8.47%)	
	Cardiovascular disease	4 (50%)	2 (25%)	2 (25%)	0 (0%)	0 (0%)	8 (2.19%)	
	Diabetes mellitus	8 (44.44%)	8 (44.44%)	2 (11.11%)	0 (0%)	0 (0%)	18 (4.92%)	
	Hypothyroidism	12 (37.5%)	17 (53.13%)	3 (9.38%)	0 (0%)	0 (0%)	32 (8.74%)	
	Asthma and respiratory disease	0 (0%)	4 (66.67%)	0 (0%)	2 (33.33%)	0 (0%)	6 (1.64%)	
	Chronic constipation	0 (0%)	3 (100%)	0 (0%)	0 (0%)	0 (0%)	3 (0.82%)	
	Rheumatoid disease	0 (0%)	1 (100%)	0 (0%)	0 (0%)	0 (0%)	1 (0.27%)	
	Other	4 (50%)	4 (50%)	0 (0%)	0 (0%)	0 (0%)	8 (2.19%)	
History of obesity-related health problems (sleep breathing disorder)	Yes, but had improved and disappeared post-surgically	52 (27.66%)	86 (45.74%)	37 (19.68%)	8 (4.26%)	5 (2.66%)	188 (51.37%)	0.029
	Yes, but unchanged compared to the preoperative time	4 (16.67%)	12 (50%)	8 (33.33%)	0 (0%)	0 (0%)	24 (6.56%)	
	No, I did not complain of disturbance during sleep	61 (39.61%)	65 (42.21%)	16 (10.39%)	9 (5.84%)	3 (1.95%)	154 (42.08%)	
Esophageal reflux	Yes, but had improved and disappeared post-surgically	49 (34.27%)	67 (46.85%)	20 (13.99%)	6 (4.2%)	1 (0.7%)	143 (39.07%)	0.001
	Yes, but unchanged compared to the preoperative time	14 (23.33%)	26 (43.33%)	12 (20%)	4 (6.67%)	4 (6.67%)	60 (16.39%)	
	Yes, worsened	6 (21.43%)	9 (32.14%)	10 (35.71%)	0 (0%)	3 (10.71%)	28 (7.65%)	
	No, I did not complain of esophageal reflux	48 (35.56%)	61 (45.19%)	19 (14.07%)	7 (5.19%)	0 (0%)	135 (36.89%)	
Body mass level before obesity surgery	30%-35%	58 (33.14%)	85 (48.57%)	20 (11.43%)	10 (5.71%)	2 (1.14%)	175 (47.81%)	0
	35%-40%	20 (17.39%)	60 (52.17%)	28 (24.35%)	3 (2.61%)	4 (3.48%)	115 (31.42%)	
	>40%	39 (51.32%)	18 (23.68%)	13 (17.11%)	4 (5.26%)	2 (2.63%)	76 (20.77%)	
Postoperative body mass level for obesity	<20%	43 (40.95%)	42 (40%)	13 (12.38%)	4 (3.81%)	3 (2.86%)	105 (28.69%)	0
	20%-25%	28 (23.14%)	58 (47.93%)	29 (23.97%)	6 (4.96%)	0 (0%)	121 (33.06%)	
	25%-30%	15 (21.13%)	35 (49.3%)	14 (19.72%)	5 (7.04%)	2 (2.82%)	71 (19.4%)	
	30%-35%	29 (54.72%)	15 (28.3%)	4 (7.55%)	2 (3.77%)	3 (5.66%)	53 (14.48%)	
	35%-40%	2 (12.5%)	13 (81.25%)	1 (6.25%)	0 (0%)	0 (0%)	16 (4.37%)	
Improving body mass index after surgery for obesity	Not improved	8 (34.78%)	6 (26.09%)	5 (21.74%)	4 (17.39%)	0 (0%)	23 (6.28%)	0.022
	Improved	109 (31.78%)	157 (45.77%)	56 (16.33%)	13 (3.79%)	8 (2.33%)	343 (93.72%)	
Type of bariatric surgery performed	Gastric sleeve surgery	94 (30.03%)	148 (47.28%)	52 (16.61%)	11 (3.51%)	8 (2.56%)	313 (85.52%)	0.009
	Gastric bypass surgery	23 (43.4%)	15 (28.3%)	9 (16.98%)	6 (11.32%)	0 (0%)	53 (14.48%)	
When was bariatric surgery performed?	<6 months	39 (50.65%)	31 (40.26%)	2 (2.6%)	5 (6.49%)	0 (0%)	77 (21.04%)	0
	6 months-1 year	15 (20.55%)	33 (45.21%)	19 (26.03%)	2 (2.74%)	4 (5.48%)	73 (19.95%)	
	1-2 years	14 (15.05%)	49 (52.69%)	22 (23.66%)	5 (5.38%)	3 (3.23%)	93 (25.41%)	
	2-3 years	18 (39.13%)	19 (41.3%)	6 (13.04%)	2 (4.35%)	1 (2.17%)	46 (12.57%)	
	3-4 years	3 (14.29%)	13 (61.9%)	2 (9.52%)	3 (14.29%)	0 (0%)	21 (5.74%)	
	>4 years	28 (50%)	18 (32.14%)	10 (17.86%)	0 (0%)	0 (0%)	56 (15.3%)	
Did you experience any problems/complications related to bariatric surgery? (n = 360)	No	102 (36.04%)	130 (45.94%)	38 (13.43%)	9 (3.18%)	4 (1.41%)	283 (77.32%)	0
	Yes	15 (19.48%)	30 (38.96%)	21 (27.27%)	7 (9.09%)	4 (5.19%)	77 (21.04%)	
Did you visit a psychiatrist's office before surgery?	No	94 (32.75%)	129 (44.95%)	44 (15.33%)	15 (5.23%)	5 (1.74%)	287 (78.42%)	0.42
	Yes	23 (29.11%)	34 (43.04%)	17 (21.52%)	2 (2.53%)	3 (3.8%)	79 (21.58%)	
If the answer is yes, when do you visit the psychiatrist's clinic? (n = 79)	Visited but cannot remember when	18 (54.55%)	15 (45.45%)	0 (0%)	0 (0%)	0 (0%)	33 (9.02%)	0.024
	Yes, in less than one year	2 (8.33%)	9 (37.5%)	11 (45.83%)	2 (8.33%)	0 (0%)	24 (6.56%)	
	Yes, 1-3 years before	0 (0%)	7 (58.33%)	5 (41.67%)	0 (0%)	0 (0%)	12 (3.28%)	
	Yes, four years before surgery	3 (30%)	3 (30%)	1 (10%)	0 (0%)	3 (30%)	10 (2.73%)	
	No	94 (32.75%)	129 (44.95%)	44 (15.33%)	15 (5.23%)	5 (1.74%)	287 (78.42%)	
Have you been diagnosed with any mental illness?	No	106 (33.23%)	147 (46.08%)	46 (14.42%)	12 (3.76%)	8 (2.51%)	319 (87.16%)	0.004
	Yes	11 (23.4%)	16 (34.04%)	15 (31.91%)	5 (10.64%)	0 (0%)	47 (12.84%)	
If the answer is yes, what psychological diagnoses have you been diagnosed with? (n = 47)	Panic attacks and social phobia	3 (30%)	2 (20%)	5 (50%)	0 (0%)	0 (0%)	10 (2.73%)	0.001
	Personality disorders	0 (0%)	6 (54.55%)	3 (27.27%)	2 (18.18%)	0 (0%)	11 (3.01%)	
	Other psychiatric illness	4 (26.67%)	3 (20%)	5 (33.33%)	3 (20%)	0 (0%)	15 (4.1%)	
	Obsessive disorder	4 (57.14%)	1 (14.29%)	2 (28.57%)	0 (0%)	0 (0%)	7 (1.91%)	
	Schizophrenia	0 (0%)	4 (100%)	0 (0%)	0 (0%)	0 (0%)	4 (1.09%)	
Are there any close family members who have been diagnosed with a psychological/mental illness?	No	103 (34.92%)	131 (44.41%)	46 (15.59%)	12 (4.07%)	3 (1.02%)	295 (80.6%)	0.003
	Yes	14 (19.72%)	32 (45.07%)	15 (21.13%)	5 (7.04%)	5 (7.04%)	71 (19.4%)	
If the answer is yes, what psychiatric diagnosis do they have? (n = 71)	Panic attacks	1 (5.56%)	6 (33.33%)	6 (33.33%)	0 (0%)	5 (27.78%)	18 (4.92%)	0.038
	Other psychiatric illness	7 (41.18%)	4 (23.53%)	3 (17.65%)	3 (17.65%)	0 (0%)	17 (4.64%)	
	Personality disorder	0 (0%)	6 (100%)	0 (0%)	0 (0%)	0 (0%)	6 (1.64%)	
	Obsessive-compulsive disorder	6 (54.55%)	0 (0%)	5 (45.45%)	0 (0%)	0 (0%)	11 (3.01%)	
	Schizophrenia	0 (0%)	16 (84.21%)	1 (5.26%)	2 (10.53%)	0 (0%)	19 (5.19%)	
Have you used medications or illegal drugs before?	No	111 (32.36%)	157 (45.77%)	55 (16.03%)	15 (4.37%)	5 (1.46%)	343 (93.72%)	0.002
	Yes, frequently	6 (26.09%)	6 (26.09%)	6 (26.09%)	2 (8.7%)	3 (13.04%)	23 (6.28%)	
Have you recently encountered family, social, economic, or work problems?	No	82 (46.07%)	81 (45.51%)	12 (6.74%)	3 (1.69%)	0 (0%)	178 (48.63%)	0
	Yes	35 (18.62%)	82 (43.62%)	49 (26.06%)	14 (7.45%)	8 (4.26%)	188 (51.37%)	

Overall, these findings underscore the complex interplay between various clinical and demographic factors and depression severity among bariatric surgery patients. Addressing these factors comprehensively in clinical assessments and interventions may contribute to improved mental health outcomes post-surgery.

## Discussion

The prevalence and consequences of depression after bariatric surgery among adults in Saudi Arabia are significant. Our study found the prevalence of depression at 23.50% in post-bariatric adult patients in Saudi Arabia. This is significantly higher than the previously reported data. According to Alyahya and Alnujaidi [1], the prevalence of post-bariatric surgery depression stands at 15.3%, impacting as many as 64.9% of patients. Alabi et al. [17] found that nearly half of people who are candidates for bariatric surgery experience some level of depression, which notably improves shortly after surgery and remains stable during the initial year. However, Martens et al. [18] observed that post-bariatric depression prevalence escalates after the first year, reaching 8.7% by the second year following surgery. This study also found that prior psychiatric consultations did not significantly influence depression severity among bariatric surgery patients. However, the timing of these visits was associated with higher levels of depression, suggesting that timely intervention and support may be beneficial. Furthermore, participants with diagnosed mental illnesses and those with family members diagnosed with psychiatric illnesses exhibited higher levels of depression severity, emphasizing the importance of addressing comorbid psychiatric conditions and considering familial influences on mental health outcomes in this population. Additionally, the study revealed a significant association between substance use behaviors and depression severity, as well as psychosocial stressors such as recent family, social, economic, or work-related problems. Psychosocial stressors such as recent family, social, economic, or work-related problems have been extensively studied as predictors of depression and anxiety. Research has shown that exposure to stressors such as job insecurity, financial instability, and social and relational issues are strong predictors of depression and anxiety symptoms among young adults [19]. Moreover, economic stressors have been associated with higher levels of depressive and anxiety symptoms, with different age cohorts manifesting varying levels of these symptoms [20].

These findings highlight the need for comprehensive assessments and interventions that address not only the biological and surgical aspects of bariatric surgery but also the psychological and social factors that can impact mental health outcomes. In conclusion, it is crucial for healthcare providers to recognize and address the prevalence and consequences of depression after bariatric surgery among adults in Saudi Arabia. This includes identifying and providing appropriate support for individuals with prior psychiatric consultations, addressing comorbid mental illnesses and familial influences, and offering interventions that address substance use behaviors and psychosocial stressors. Furthermore, community-based services and complementary therapies were found to be significantly related to improvements in psychological health and depression scores among individuals experiencing additional life stresses.

However, the study has some limitations. These include a small sample size, reliance on self-report measures for assessing depression severity, and a cross-sectional design that limits the ability to establish causality. Additionally, the study was conducted in a specific population (adults in Saudi Arabia) and may not be generalizable to other populations or settings. The study also did not explore other potential factors that might contribute to depression after bariatric surgery, such as changes in suboptimal weight loss after the surgery and body dissatisfaction. Switzer et al. [21] reported a strong association between rebound weight gain and depressive manifestations after bariatric surgery. Overall, the study highlights the importance of addressing and providing support for mental health issues, particularly depression, among individuals who have undergone bariatric surgery.

## Conclusions

The high prevalence of depression after bariatric surgery among adults in Saudi Arabia indicates the urgent need for comprehensive mental health evaluation and support for individuals undergoing this procedure. The substantial proportion of participants experiencing depression underscores the importance of integrating mental healthcare into bariatric surgery programs. Additionally, it highlights the necessity of implementing strategies to identify and address depression in the postoperative period to optimize the overall well-being and long-term success of bariatric surgery patients. The significant consequence of depression after bariatric surgery emphasizes the imperative for healthcare providers to prioritize mental health screening, intervention, and follow-up care within the bariatric surgery framework. Addressing the psychological impact of bariatric surgery can potentially improve outcomes and enhance the overall quality of life for individuals undergoing this weight loss intervention. Further research and multifaceted interventions are warranted to better understand the underlying mechanisms and risk factors associated with depression after bariatric surgery and to develop tailored approaches for prevention and management. Integrating mental health support as an integral component of bariatric care may lead to improved patient experiences and long-term physical and psychological well-being.
